# Preoperative systemic inflammatory response index predicts the prognosis of patients with hepatocellular carcinoma after liver transplantation

**DOI:** 10.3389/fimmu.2023.1118053

**Published:** 2023-03-27

**Authors:** Songping Cui, Shuang Cao, Qing Chen, Qiang He, Ren Lang

**Affiliations:** Department of Hepatobiliary and Pancreaticosplenic Surgery, Beijing ChaoYang Hospital, Capital Medical University, Beijing, China

**Keywords:** hepatocellular carcinoma, liver transplantation, biomarkers, systemic inflammatory response index, prognosis

## Abstract

**Background:**

Preoperative inflammatory status plays an important role in the prognosis of malignancy. We sought to explore the value of preoperative inflammatory biomarkers in predicting long-term outcomes of liver transplantation (LT) in patients with hepatocellular carcinoma (HCC).

**Method:**

Patients who underwent LT for HCC in our hospital between January 2010 and June 2020 were included in this study. Demographic, clinical, laboratory, and outcome data were obtained. The area under the curve (AUC) of the receiver operating characteristic curve was used to evaluate the predictive value of inflammatory biomarkers. The effectiveness of inflammatory biomarkers in predicting outcomes was analyzed by univariate and multivariate Cox proportional hazards analyses.

**Results:**

A total of 218 patients were included in the study, with a mean age of 53.9 ± 8.5 years. The AUC of neutrophil-to-lymphocyte ratio (NLR), platelet-to-lymphocyte ratio (PLR), monocyte-to-lymphocyte ratio (MLR), systemic immune inflammation index (SII), and systemic inflammatory response index (SIRI) for overall survival (OS) were 0.741, 0.731, 0.756, 0.746, and 0.749, respectively. Cox proportional hazards model indicated that SIRI > 1.25 was independently associated with low OS [hazard ratio (HR) = 2.258, P = 0.024]. PLR > 82.15 and SIRI > 0.95 were independently associated with low disease-free survival (HR = 1.492, P = 0.015; and HR = 1.732, P = 0.008, respectively). In the survival analysis, the prognosis of patients with high preoperative SIRI and PLR was significantly worse (P < 0.001).

**Conclusion:**

SIRI and PLR were useful prognostic markers for predicting patients with HCC after LT.

## Introduction

Hepatocellular carcinoma (HCC) is the most common pathological type of primary liver cancer, and the incidence is increasing year by year (1, 2). It is also an important factor of cancer-related death (3, 4). For patients with early-stage HCC, liver transplantation (LT) is undoubtedly one of the most effective forms of treatment. LT can radically remove the carcinoma and also cure liver disease such as cirrhosis, which removes major risk factors for new-onset tumors. Therefore, of all the treatments, LT is the most likely to cure the patient (5–7). Although patients with HCC were screened according to selection criteria before receiving LT, about 10%–20% of patients still experience recurrence after LT (8, 9). This also severely limits the long-term survival of these patients. Therefore, clinicians need to screen out patients who will have better survival benefits after LT to make more efficient use of scarce donor liver sources.

Recently, clinical biomarkers based on laboratory results help to objectively assess the patient’s status and have predictive value for some patient outcomes (10–13). These include several inflammatory and immune scores, such as platelet-to-lymphocyte ratio (PLR), systemic immune inflammation index (SII), neutrophil-to-lymphocyte ratio (NLR), systemic inflammatory response index (SIRI), and monocyte-to-lymphocyte ratio (MLR). NLR, PLR, and MLR reflect immune function and inflammatory states, which are also useful for predicting tumor recurrence and early death (11–17). SII, calculated from platelet, neutrophil, and lymphocyte counts, is a strong predictor of poor prognosis for patients with HCC and helps physicians make clinical decisions (18–20). SIRI may be effective in reflecting the dynamics of inflammation and immune status, predicting prognosis after resection of HCC and response to systemic treatment for HCC (21–23).

We conducted this study to determine the utility of preoperative inflammatory biomarkers in predicting long-term outcomes in patients with HCC after LT.

## Materials and methods

### Study population

**Figure 1:**
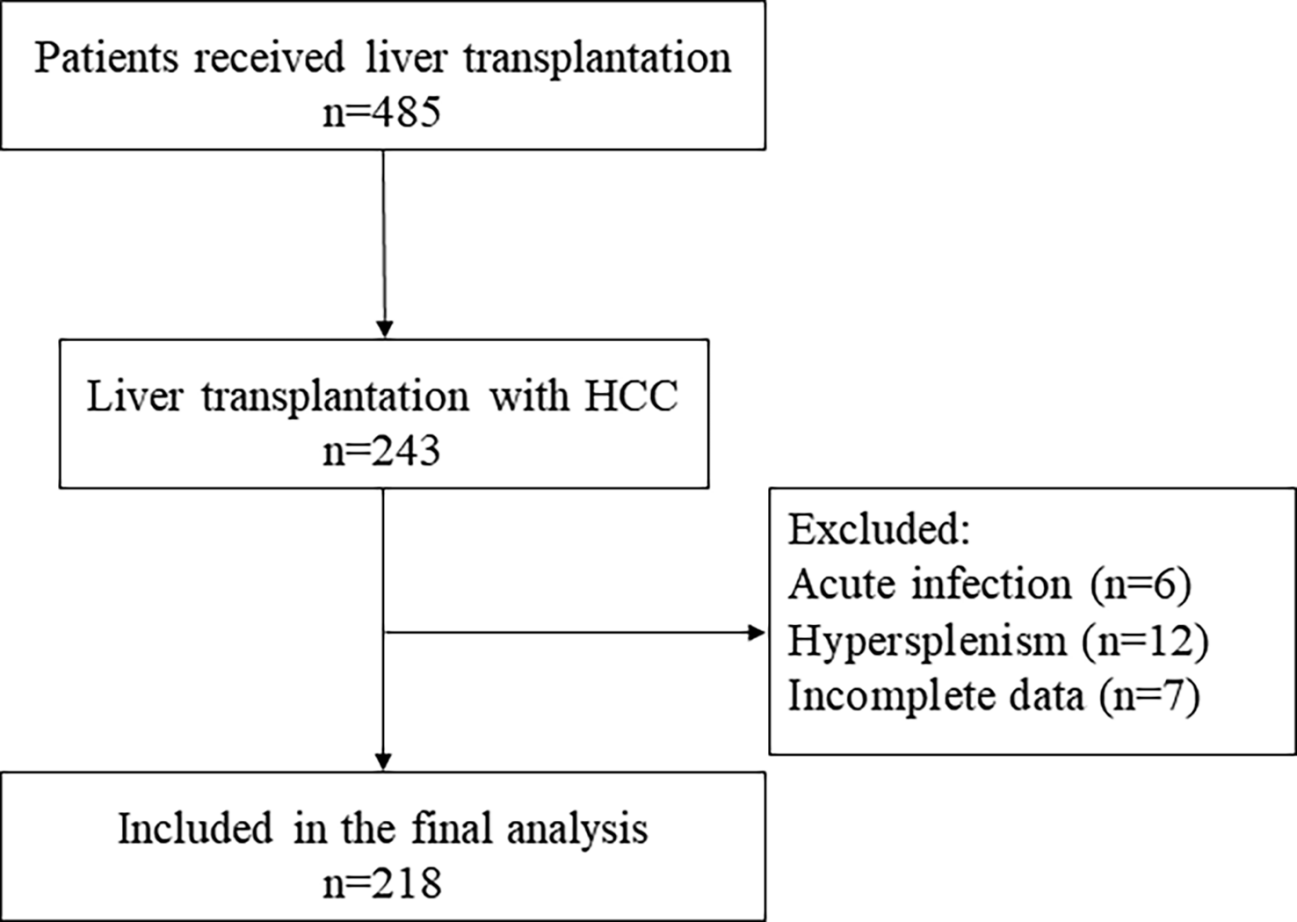
Flowchart of the study cohort. HCC, hepatocellular carcinoma.

We retrospectively collected patients who received LT for HCC in our hospital between January 2010 and June 2020. The selection of the study population is represented by a flowchart (**Figure 1**).

Exclusion criteria are as follows: (1) combined acute infection, (2) preoperative diagnosis of hypersplenism, and (3) incomplete data.

### Data collection and follow-up

We applied electronic medical record system to collect the following data: demographic and laboratory characteristics. All enrolled patients had venous blood samples taken within 24 h of admission and performed complete blood count analysis. About biomarkers, NLR was the ratio of neutrophil count to lymphocyte count, PLR was the ratio of platelet count to lymphocyte count, SII was defined as platelet count * neutrophil count/lymphocyte count, MLR was the ratio of monocyte count to lymphocyte count, and SIRI was defined as monocyte count * neutrophil count/lymphocyte count.

We followed all patients by telephone and outpatient or inpatient observation until June 2022 or death. Follow-ups were performed every 3 months for 2 years after surgery and every 6 months thereafter. If there was a suspicious lesion in the liver or lungs, then biopsy was performed to determine whether it was recurrence or metastasis. The time recurrence started was defined as the time when AFP levels started to rise. LT to death or last observation was defined as overall survival (OS), and LT to recurrence was defined as disease-free survival (DFS).

### Statistical analysis

Continuous variables were compared between groups by either the Student’s t-test or the Mann-Whitney U-test. Categorical variables were compared between groups by Pearson’s chi-square test or Fisher’s exact test. The optimal cutoff value was determined by receiver operating characteristic (ROC) curve. Independent prognostic factors related to OS and DFS were identified by Cox regression models. Variables were included in multivariate analysis if P < 0.2 in univariate analysis. Kaplan–Meier curves with the log-rank test were used to survival analysis. Statistical significance was defined as two-sided P < 0.05. All statistical analyses were performed using SPSS 26.0 and GraphPad Prism 8.0.

## Results

### Characteristics of patients

Flowchart shows the included and excluded patients (**Figure 1**). A total of 218 patients were recruited. The mean age of all patients was 53.9 ± 8.5 years, in which 90.4% are men (197 of 218). The median follow-up was 39.4 months. Survival outcomes at 2 years of follow-up determined patient grouping.

The baseline characteristics are shown in **Table 1**. Compared to the non-survival group, the survival group had a significantly higher body mass index (BMI) of 25.0 ± 3.9 (P = 0.014). There were no significant differences in age and sex (P > 0.05). Common comorbidities such as diabetes, hypertension, hepatic encephalopathy, and HBV positivity were also not significantly different between groups (P > 0.05). In the pathological information, tumor number, total tumor size, largest tumor size, and degree of tumor differentiation were significantly different between groups (P < 0.001).

**Table 1:** Demographic and baseline characteristics of the total cohort.

Variables	Total (n = 218)	Survival (n = 163)	Non-survival (n = 55)	P
Age	53.9 ± 8.5	54.0 ± 8.4	53.7 ± 8.7	0.811
Gender (male)	197 (90.4)	147 (90.2)	50 (90.9)	0.875
BMI	24.7 ± 3.6	25.0 ± 3.9	23.7 ± 2.7	**0.014**
Diabetes	53 (24.3)	38 (23.3)	15 (27.3)	0.554
Hypertension	46 (21.1)	35 (21.8)	11 (20.0)	0.817
Hepatic encephalopathy	71 (32.6)	48 (29.4)	23 (41.8)	0.091
HBV positivity (n,%)	182 (83.5)	138 (84.7)	44 (80.0)	0.637
White blood cell (×10^9^/L)	4.5 ± 2.3	4.2 ± 2.0	5.1 ± 2.8	**0.018**
Neutrophil (×10^9^/L)	2.9 ± 1.8	2.7 ± 1.5	3.5 ± 2.3	**0.008**
Lymphocyte (×10^9^/L)	1.0 ± 0.7	0.8 ± 0.6	1.1 ± 0.7	**0.003**
Monocyte (×10^9^/L)	0.36 ± 0.26	0.33 ± 0.21	0.43 ± 0.34	**0.026**
Platelet (×10^9^/L)	109.7 ± 86.3	104.8 ± 88.8	121.6 ± 79.5	0.212
Albumin (g/L)	35.7 ± 6.1	35.6 ± 6.2	35.9 ± 5.7	0.691
Serum creatinine (μmol/L)	71.6 ± 39.3	72.1 ± 45.2	70.5 ± 19.2	0.791
Total bilirubin (μmol/L)	26.3 (32.8)	24.2 (33.9)	27.4 (31.6)	0.919
AFP (ng/ml)	18.2 (112.3)	9.6 (56.3)	87.6 (881.4)	**0.034**
MELD score	9.9 ± 4.0	9.9 ± 4.2	9.8 ± 3.3	0.870
Tumor number (>3)	45 (20.6)	24 (14.7)	21 (38.2)	**<0.001**
Largest tumor size (cm)	3.5 (3.5)	3.0 (2.1)	5.0 (5.7)	**<0.001**
Total tumor size (cm)	5.0 (5.0)	4.0 (4.5)	8.0 (6.6)	**<0.001**
Differentiation (1–2)	177 (81.2)	123 (75.5)	54 (98.2)	**<0.001**
Lymph node staging (1–2)	6 (2.8)	4 (2.5)	2 (3.6)	**0.644**
NLR	2.7 (2.9)	2.2 (2.1)	4.5 (4.8)	**0.001**
PLR	94.4 (84.3)	81.6 (57.5)	157.9 (250.6)	**<0.001**
MLR	0.3 (0.4)	0.3 (0.2)	0.6 (0.4)	**<0.001**
SII	243.5 (396.3)	198.9 (224.9)	544.3 (720.4)	**<0.001**
SIRI	0.8 (1.1)	0.7 (0.7)	1.6 (02.9)	**<0.001**

BMI, body mass index; HBV, hepatitis B virus; AFP, α-fetoprotein; NLR, neutrophil-to-lymphocyte ratio; PLR, platelet-to-lymphocyte ratio; MLR, monocyte-to-lymphocyte ratio; SII, systemic immune inflammation index; SIRI, systemic inflammatory response index. Bold indicates that the P value is statistically different (P < 0.05).

Compared with the survival group, the white blood cells, neutrophil, lymphocyte, monocyte counts, and AFP of the non-survival group increased significantly (P = 0.018, 0.008, 0.003, 0.026, and 0.034, respectively). There were significant differences in preoperative NLR, PLR, MLR, SII, and SIRI between groups (P = 0.001, < 0.001, < 0.001, < 0.001, and < 0.001, respectively).

### Preoperative inflammatory biomarkers and prognosis

**Figure 2:**
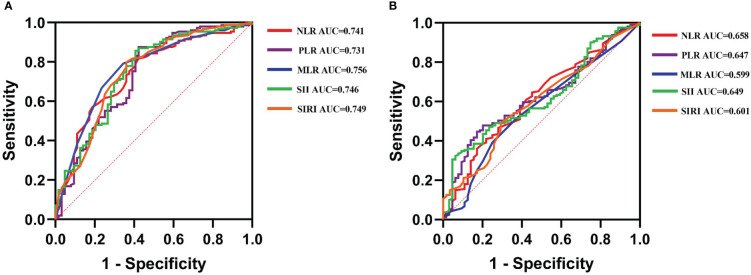
The receiver operating characteristic (ROC) curves explore the value of preoperative inflammatory biomarkers in predicting the long-time prognosis in liver transplantation (LT) for hepatocellular carcinoma (HCC). **(A)** The value of ROC in predicting overall survival in LT for HCC. **(B)** The value of ROC in predicting disease-free survival in LT for HCC.

The ROC curves were used for exploring the correlation between preoperative inflammatory biomarkers and outcomes after LT (**Figures 2A, B**). The optimal cutoff values for NLR, PLR, MLR, SII, and SIRI to predict postoperative OS were 4.1, 82.15, 0.45, 179.8, and 1.25, respectively [the area under the curve (AUC) = 0.741, 0.731, 0.756, 0.746, and 0.749, respectively; **Supplementary Table 1**]. For DFS, the optimal cutoff values were 2.35, 82.15, 0.45, 86.3, and 0.95, respectively (AUC = 0.658, 0.647, 0.599, 0.649, and 0.601, respectively; **Supplementary Table 2**).

**Tables 2** and **3** show the results of Cox regression analysis. Multivariate Cox proportional hazards regression analysis showed that BMI (>25.55), AFP (>73.2), tumor number (>3), differentiation (1–2), and SIRI (>1.25) were independent related factors for OS [hazard ratio (HR) = 0.924, P = 0.029; HR = 2.376, P = 0.002; HR = 2.193, P = 0.002; HR = 3.006, P = 0.024; and HR = 2.258, P = 0.024, respectively] (**Table 2**). AFP (>73.2), tumor number (>3), differentiation (1–2), PLR (>82.15), and SIRI (>0.95) were the independent risk factors for DFS (HR = 1.626, P = 0.005; HR = 1.861, P = 0.001; HR = 2.435, P = 0.001; HR = 1.492, P = 0.015; and HR = 1.732, P = 0.008, respectively) (**Table 3**).

**Table 2:** Cox regression analysis of the effects of clinicopathological factors on the overall survival of patients.

Variables	Univariate Cox regression		Multivariate Cox regression	
	HR (95% CI)	P	HR (95% CI)	P
BMI (>25.55)	0.915 (0.853–0.982)	**0.013**	0.924 (0.861–0.992)	**0.029**
Hepatic encephalopathy	1.726 (1.054–2.827)	**0.030**	1.577 (0.916–2.712)	0.100
AFP (>73.2)	2.853 (1.342–4.339)	**<0.001**	2.376 (1.386–4.075)	**0.002**
Tumor number (>3)	2.872 (1.736–4.751)	**<0.001**	2.193 (1.277–3.767)	**0.002**
Differentiation (1–2)	3.612 (2.513–5.241)	**0.004**	3.066 (2.231–5.022)	**0.024**
NLR (>4.1)	1.074 (1.043–1.106)	**<0.001**	0.997 (0.923–1.077)	0.942
PLR (>82.15)	1.036 (1.012–1.156)	**<0.001**	1.002 (0.998–1.007)	0.336
MLR (>0.45)	2.194 (1.631–2.953)	**<0.001**	0.626 (0.151–2.600)	0.519
SII (>179.8)	1.159 (1.085–1.342)	**<0.001**	1.000 (0.999–1.001)	0.766
SIRI (>1.25)	2.076 (1.533–3.121)	**<0.001**	2.258 (1.530–3.535)	**0.024**

BMI, body mass index; AFP, α-fetoprotein; NLR, neutrophil-to-lymphocyte ratio; PLR, platelet-to-lymphocyte ratio; MLR, monocyte-to-lymphocyte ratio; SII, systemic immune inflammation index; SIRI, systemic inflammatory response index; HR, hazard ratio; CI, confidence interval. Bold indicates that the P value is statistically different (P < 0.05).

**Table 3:** Cox regression analysis of the effects of clinicopathological factors on the disease-free survival of patients.

Variables	Univariate Cox regression		Multivariate Cox regression	
	HR (95% CI)	P	HR (95% CI)	P
BMI (>25.55)	1.005 (0.837–1.005)	0.837	–	
Hepatic encephalopathy	1.296 (0.922–1.823)	0.236	–	
AFP (>73.2)	2.270 (1.651–3.123)	**<0.001**	1.626 (1.155–2.288)	**0.005**
Tumor number(>3)	1.983 (1.388–2.833)	**<0.001**	1.861 (1.281–2.704)	**0.001**
Differentiation (1–2)	2.418 (1.527–3.830)	**<0.001**	2.435 (1.460–4.060)	**0.001**
NLR (>2.35)	1.057 (1.029–1.085)	**<0.001**	1.019 (0.956–1.086)	0.563
PLR (>82.15)	1.706 (1.240–2.349)	**0.001**	1.492 (1.142–1.942)	**0.015**
MLR (>0.45)	1.627 (1.222–2.165)	**0.001**	0.527 (0.224–1.236)	0.141
SII (>86.3)	2.548 (1.517–4.278)	**<0.001**	1.000 (0.999–1.000)	0.603
SIRI (>0.95)	1.985 (1.455–2.707)	**0.002**	1.732 (1.240–2.433)	**0.008**

BMI, body mass index; AFP, α-fetoprotein; NLR, neutrophil-to-lymphocyte ratio; PLR, platelet-to-lymphocyte ratio; MLR, monocyte-to-lymphocyte ratio; SII, systemic immune inflammation index; SIRI, systemic inflammatory response index; HR, hazard ratio; CI, confidence interval. Bold indicates that the P value is statistically different (P < 0.05).

**Figure 3:**
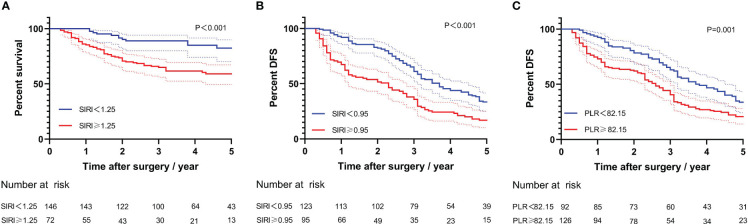
Kaplan–Meier curves for long-time prognosis by PLR and SIRI. **(A)** Kaplan–Meier curves for overall survival by SIRI. **(B)** Kaplan–Meier curves for disease-free survival by SIRI. **(C)** Kaplan–Meier curves for disease-free survival by PLR.

Kaplan–Meier survival analysis for long-term prognosis was shown in **Figures 3A–C**. Compared with the high-SIRI group, the 5-year OS rate and DFS rate of the low-SIRI group were significantly higher (40.2% *vs*. 81.9%, log-rank test, P < 0.001; 16.8% *vs*. 33.3%, log-rank test, P < 0.001; **Figures 3A, B**). In addition, compared with the high-PLR group, the 5-year DFS rate of the low-PLR group was significantly higher (20.6% *vs*. 33.7%, log-rank test, P = 0.001; **Figure 3C**).

## Discussion

Our study found that inflammatory biomarkers such as PLR and SIRI were significantly related to long-term prognosis after LT for HCC. High SIRI and high PLR were the independent risk factors for poor prognosis, particularly SIRI, which was clearly associated with both OS and DFS.

Over the past 10 years, NLR has received increasing attention as an emerging disease biomarker. NLR is the ratio of neutrophil count to lymphocyte count, combining the innate immune response and acquired immunity in the immune system (24). NLR has been shown to be critical in sepsis, pneumonia, COVID-19, cardiovascular disease, cancer, and other diseases and is independently related to prognosis (25–31). In terms of cancers, it is pointed out that inflammation contributes to the development and progression of cancers (32). The correlation between NLR and HCC prognosis has also been actively explored. Studies found that patients with HCC with low NLR were more likely to have a better long-term prognosis (33–35). Moreover, NLR also has clear correlation with long-term prognosis after surgical resection, LT, transarterial chemoembolization, and radiofrequency ablation, with HRs ranging from 1.16 to 4.22 (33, 36–39).

PLR, MLR, and SII have been widely used in predicting the death and recurrence of HCC (17–20, 40–44). This study showed a significant correlation between high preoperative PLR and low DFS in patients with HCC receiving LT. This is also consistent with some previous findings (45–47). The correlation of PLR with tumor characteristics may play an important role. High PLR has been shown to be associated with larger tumor size and lymph node metastasis (48, 49). As for HCC, studies have shown that high PLR means a high likelihood of advanced tumor staging and aggressive tumor phenotype (45). On the one hand, tumor cells can activate platelets, and activated platelets will directly adhere to tumor cells, helping tumor cells to escape immunity (50, 51). On the other hand, activated platelets can also promote tumor angiogenesis and development by expressing cytokines (52, 53). In addition, activated platelets can cause cancer-associated thrombosis, resulting in a poor prognosis (54, 55). Lymphocytes are also an important part of anti-tumor immunity, which can secrete cytokines to activate anti-tumor immunity and directly kill tumor cells, thereby inhibiting the proliferation and migration of tumor cells. Therefore, when the number of lymphocytes in the peripheral blood decreases, the body’s defenses against cancer cells are also weakened, which can lead to tumor progression and recurrence (56).

SIRI is calculated from lymphocyte, neutrophil, and monocyte counts, and its predictive value for malignancy prognosis has been demonstrated (57–60). Nevertheless, SIRI still does not receive enough attention in patients with HCC with LT. This study showed a clear correlation between high SIRI and poor long-term outcomes in this population. In addition to the lymphocytes mentioned above, neutrophils and monocytes also play critical roles in the development of tumors. At present, the mechanism has not been fully clarified. Tumor cells can secrete cytokines to stimulate the proliferation and differentiation of neutrophils and promote the increase of circulating neutrophil levels (61). High levels of circulating neutrophils lead to decreased function of T cells and lymphocytes, leading to weakened immune function (62, 63). Clinically, elevated peripheral neutrophil counts are clearly related to high polymorphonuclear neutrophil myeloid-derived suppressor cells (PMN-MDSCs), resulting in poor prognosis for patients with HCC (64). PMN-MDSCs have immunosuppressive ability, and, in HCC, they can regulate arginase-1 and reactive oxygen species to inhibit T cells (65, 66). Notably, it is pointed out that PMN-MDSCs and tumor-associated neutrophils have some similarities in origin, morphology, and molecular phenotype and also have some functional overlap (67). Similarly, previous studies have confirmed that the number of circulating monocytes determines the number of tumor-associated macrophages (68–70). As for TAM, it is able to induce apoptosis of T cells with anticancer functions and promote tumor angiogenesis, thereby promoting tumor growth, invasion, and migration, resulting in a poor prognosis (71, 72).

There were several limitations in our study. First, the study was retrospective, single-center that would skew the results. The results still need to be further validated in multi-center prospective studies. Second, preoperative inflammatory biomarkers were affected by many factors, such as heavy bleeding and instrument errors, making the measurement results inaccurate. Last, this study did not take repeated measurements at different time points to observe the dynamics of these biomarkers.

## Conclusion

Elevated preoperative SIRI and PLR were the independent risk factors for poor prognosis in LT for HCC. This result will help clinicians to screen out patients with better prognosis before surgery and to provide some reference for decision-making on comprehensive treatment after surgery.

## Data availability statement

The original contributions presented in the study are included in the article/**Supplementary Material**. Further inquiries can be directed to the corresponding author.

## Ethics statement

The study involving human participants were reviewed and approved by Beijing Chaoyang Hospital ethics committee (No.2020-D.304). The patients/participants provided their written informed consent to participate in this study.

## Author contributions

SPC and SC conceived and designed the study. SC and QC collected the data. SC and QC analyzed the data. SPC wrote the original draft. QH and RL revised the manuscript. RL administered and supervised the study. All authors contributed to the article and approved the submitted version.
