# Supplementary material for: Preoperative systemic inflammatory response index predicts the prognosis of patients with hepatocellular carcinoma after liver transplantation
Source: Front Immunol. 2023 Mar 27;14:1118053. doi: 10.3389/fimmu.2023.1118053 (PMC10083266; doi:10.3389/fimmu.2023.1118053)
Supplement: Supplementary file 1 [file Table_1.docx]

**Supplementary Table 1** Receiver operating characteristic curve analysis for overall survival

| **Variables** | **cut-off value** | **AUC** | **sensitivity** | **specificity** |
| --- | --- | --- | --- | --- |
| NLR | 4.1 | 0.741 | 0.818 | 0.578 |
| PLR | 82.15 | 0.731 | 0.507 | 0.781 |
| MLR | 0.45 | 0.756 | 0.792 | 0.656 |
| SII | 179.8 | 0.746 | 0.455 | 0.813 |
| SIRI | 1.25 | 0.749 | 0.799 | 0.641 |
| BMI | 25.55 | 0.601 | 0.364 | 0.828 |
| AFP | 73.2 | 0.675 | 0.792 | 0.578 |

NLR, neutrophil to lymphocyte ratio; PLR, platelet to lymphocyte ratio; MLR, monocyte to lymphocyte ratio; SII, the ratio of platelet multiply neutrophil to lymphocyte; SIRI, the ratio of monocyte multiply neutrophil to lymphocyte; BMI, body mass index; AFP, α-fetoprotein.

**Supplementary Table 2** Receiver operating characteristic curve analysis for disease-free survival

| **Variables** | **cut-off value** | **AUC** | **sensitivity** | **specificity** |
| --- | --- | --- | --- | --- |
| NLR | 2.35 | 0.658 | 0.646 | 0.547 |
| PLR | 82.15 | 0.647 | 0.621 | 0.516 |
| MLR | 0.45 | 0.599 | 0.391 | 0.750 |
| SII | 86.3 | 0.649 | 0.901 | 0.250 |
| SIRI | 0.95 | 0.601 | 0.491 | 0.707 |
| BMI | 25.55 | 0.619 | 0.362 | 0.855 |
| AFP | 73.2 | 0.692 | 0.785 | 0.618 |

NLR, neutrophil to lymphocyte ratio; PLR, platelet to lymphocyte ratio; MLR, monocyte to lymphocyte ratio; SII, the ratio of platelet multiply neutrophil to lymphocyte; SIRI, the ratio of monocyte multiply neutrophil to lymphocyte; BMI, body mass index; AFP, α-fetoprotein
